# Astrocytes increase the activity of synaptic GluN2B NMDA receptors

**DOI:** 10.3389/fncel.2015.00117

**Published:** 2015-04-17

**Authors:** Junghyun Hahn, Xianhong Wang, Marta Margeta

**Affiliations:** Department of Pathology, University of California San FranciscoSan Francisco, CA, USA

**Keywords:** NMDA receptor, GluN2B subunit, astrocytes, synapses, protein kinase C

## Abstract

Astrocytes regulate excitatory synapse formation and surface expression of glutamate AMPA receptors (AMPARs) during development. Less is known about glial modulation of glutamate NMDA receptors (NMDARs), which mediate synaptic plasticity and regulate neuronal survival in a subunit- and subcellular localization-dependent manner. Using primary hippocampal cultures with mature synapses, we found that the density of NMDA-evoked whole-cell currents was approximately twice as large in neurons cultured in the presence of glia compared to neurons cultured alone. The glial effect was mediated by (an) astrocyte-secreted soluble factor(s), was Mg^2+^ and voltage independent, and could not be explained by a significant change in the synaptic density. Instead, we found that the peak amplitudes of total and NMDAR miniature excitatory postsynaptic currents (mEPSCs), but not AMPAR mEPSCs, were significantly larger in mixed than neuronal cultures, resulting in a decreased synaptic AMPAR/NMDAR ratio. Astrocytic modulation was restricted to synaptic NMDARs that contain the GluN2B subunit, did not involve an increase in the cell surface expression of NMDAR subunits, and was mediated by protein kinase C (PKC). Taken together, our findings indicate that astrocyte-secreted soluble factor(s) can fine-tune synaptic NMDAR activity through the PKC-mediated regulation of GluN2B NMDAR channels already localized at postsynaptic sites, presumably on a rapid time scale. Given that physiologic activation of synaptic NMDARs is neuroprotective and that an increase in the synaptic GluN2B current is associated with improved learning and memory, the astrocyte-induced potentiation of synaptic GluN2B receptor activity is likely to enhance cognitive function while simultaneously strengthening neuroprotective signaling pathways.

## Introduction

Glial cells play a critical role in the formation and function of excitatory synapses. In particular, it is well established that during development astrocyte-secreted soluble messengers promote synapse formation and surface expression of glutamate AMPA receptors (AMPARs) in both retinal ganglion cells and hippocampal neurons (Ullian et al., 2001; Christopherson et al., 2005; Xu et al., 2010; Allen et al., 2012). Much less is known about glial regulation of NMDA glutamate receptors (NMDARs), which together with AMPARs mediate excitatory postsynaptic signaling.

NMDARs are crucial for a wide range of brain functions, including synaptic plasticity and neural development (Cull-Candy and Leszkiewicz, 2004; Zito et al., 2009). NMDAR dysfunction is implicated in many neurologic and psychiatric disorders (Cull-Candy et al., 2001; Hardingham and Bading, 2003; Schwartz et al., 2012), highlighting the importance of the precise regulation of NMDAR activity. NMDAR functional diversity is controlled through two distinct mechanisms: subunit composition and subcellular localization (Paoletti et al., 2013; Sanz-Clemente et al., 2013). NMDAR channels consist of two GluN1 and two GluN2 subunits, with GluN2 subunits conferring distinct functional properties on the assembled heterotetrameric channel complexes. Hippocampal neurons primarily express GluN1/GluN2A and GluN1/GluN2B NMDARs; in comparison with GluN1/GluN2A channels, GluN1/GluN2B channels have higher glutamate and glycine sensitivity, lower open probability, and slower deactivation kinetics (Paoletti et al., 2013). In addition, GluN2A and GluN2B subunits differ in their binding affinities for various intracellular scaffolding and signaling proteins (Paoletti et al., 2013). Irrespectively of the subunit composition, NMDAR coupling to intracellular signaling cascades also depends on the receptor subcellular localization. Although non-physiologic (tonic) activation of synaptic NMDARs can lead to excitotoxicity (Parsons and Raymond, 2014), these receptors preferentially couple to neuroprotective signaling pathways when activated by physiologic (phasic) stimuli (Hardingham and Bading, 2010; Martel et al., 2012). In contrast, activation of extrasynaptic NMDARs is generally neurotoxic and has been implicated in the pathogenesis of several neurodegenerative disorders (Parsons and Raymond, 2014).

Astrocytes can activate NMDARs by secreting the NMDAR ligands glutamate and D-serine, thus directly modulating synaptic efficacy and plasticity (Haydon and Carmignoto, 2006; Lee et al., 2007; Perea and Araque, 2010; Han et al., 2013). Astrocytes were also shown to regulate the surface expression of GluN2A and GluN2B NMDARs (Fellin et al., 2009; Deng et al., 2011), but a recent study raised the possibility that these findings were an artifact of the transgenic model system used for experiments (Fujita et al., 2014). Here, we use wild-type hippocampal cultures with mature synapses to show that astrocytes secrete a soluble factor or factors that increase the activity, but not the cell surface expression, of synaptic GluN2B-containing NMDARs through a protein kinase C (PKC)-mediated mechanism. Thus, astrocytes can regulate NMDAR function in both a subunit- and localization-specific manner, without altering the trafficking of NMDAR channel subunits.

## Materials and Methods

### Cell Cultures

Mixed, neuronal, and astrocytic primary hippocampal cultures were prepared from E18–E19 embryos obtained from Sprague-Dawley rats (Charles River), as described previously (Habas et al., 2013). All procedures involving animals were approved by the UCSF Animal Care and Use Committee. Mixed cell suspension was plated at a density of 600 cells/mm^2^, either in tissue culture dishes coated with 0.1 mg/mL poly-L-lysine or on 12-mm glass cover slips (Bellco) coated with the mixture of poly-L-lysine and laminin. To obtain predominantly neuronal cultures, a subset of cultures was treated with 1 μM AraC (Sigma) to suppress glial cell growth; mixed cultures were left untreated. Mixed and neuronal cultures were grown in Neurobasal/B27 culture medium for 2–3 weeks before experiments. For astrocytic cultures, hippocampal cell suspension was plated in 75 cm^2^ tissue culture flasks and grown in DMEM/fetal bovine serum culture medium. One week after plating (when the astrocyte layer became confluent), cultures were vigorously shaken (300 rpm at 37°C) overnight to remove microglia and oligodendroglia; astrocytes were then trypsinized, re-plated into 175 cm^2^ tissue culture flasks, and maintained for another 3–4 days before being used for direct or indirect neuron-astrocyte co-cultures (Habas et al., 2013). For direct co-cultures, astrocytes were plated into DIV10 AraC-treated neuronal cultures at a density of ~120 cells/mm^2^; for indirect co-cultures, astrocytes were plated at the same density on cell culture inserts, which were then placed on top of DIV10 neuronal cultures. (The insert membrane has a pore size of 1 μm and is separated from the well bottom by 800 μm; this culture arrangement allows neurons and astrocytes to exchange soluble factors while preventing a direct cell-cell contact.). In both co-culture arrangements, the two cell types were cultured together for 4–7 days prior to experiments.

### Electrophysiology

DIV13–17 neurons were used for patch-clamp recordings. NMDA-evoked currents were recorded at −60 mV (except in I/V analysis experiments) in the external solution containing (in mM) 135 NaCl, 5 KCl, 2 CaCl_2_, 1.2 MgCl_2_, 10 HEPES, and 10 glucose, with 1 μM glycine, 10 μM bicuculline, and 1 μM TTX (~305 mOsm, pH 7.4); for 0 Mg^2+^ external solution, MgCl_2_ was replaced with glucose. The pipette solution contained (in mM) 120 CsCl, 20 TEA-Cl, 1 MgCl_2_, 1 CaCl_2_, 2.25 EGTA, 10 HEPES, 2.5 Mg_2_ATP, 1 Na_3_GTP (~298 mOsm, pH 7.4; calculated free Ca^2+^ concentration, 70 nM). The liquid junction potential, measured with 3 M KCl, was −8.8 mV and was not adjusted except for I/V and unitary conductance analyses. mEPSC recording solutions were adapted from previously published studies (Watt et al., 2000; Fu et al., 2005; Lee et al., 2007). For total excitatory mEPSC recordings, 0 Mg^2+^ external solution was supplemented with 10 μM glycine, 1 μM TTX, and 20 μM bicuculline; AP5 (100 μM) was added to isolate AMPAR-mediated mEPSCs, while 1 μM strychnine and 10 μM NBQX were added to isolate NMDAR-mediated mEPSCs. The pipette solution contained (in mM): 125 K-gluconate, 10 KCl, 10 Na-phosphocreatine, 2.25 EGTA, 2.5 Mg_2_ATP, 0.5 Na_3_GTP, 10 HEPES (~297 mOsm, pH 7.4); this pipette solution was also used for whole-cell recordings in MK-801 experiments. Drugs were purchased from Tocris Bioscience [except for bicuculline (Sigma-Aldrich) and thrombospondin (Haematologic Technologies)] and were applied through an electronically controlled, gravity-fed perfusion system with a response time of 15–20 ms (ALA Scientific); after placing a cell in the center of the visual field under 10× objective, the perfusion tip with an opening diameter of 500 μm was placed at the field edge. The pipette electrodes were pulled from borosilicate glass tubes (Sutter Instruments) and had resistances of 3.5–4.2 MΩ. Recordings were obtained using a Multiclamp 700B amplifier (Molecular Devices), with signals filtered at 1 kHz and sampled at 10 kHz using a Digidata 1440A analog-to-digital converter (Molecular Devices). All data were recorded and analyzed with pClamp 10 software (Molecular Devices).

### mEPSC Analysis

mEPSC data were analyzed using the Mini Analysis Program (Synaptosoft). Total and AMPAR mEPSC amplitudes were calculated by averaging the peak amplitudes of all individual mEPSC events recorded from a single neuron in the absence (total mEPSCs) or presence of AP5 (AMPAR mEPSCs). To obtain the average NMDAR-mediated mEPSC amplitude from the same set of recordings, the average AMPAR mEPSC trace (obtained by aligning and averaging all mEPSC events recorded from one neuron in the presence of AP5) was subtracted from the average total mEPSC trace (obtained by aligning and averaging all events recorded from the same neuron in the absence of AP5). In a separate set of recordings, NMDAR mEPSCs were recorded directly after blocking AMPAR mEPSCs with NBQX; for these experiments, NMDAR mEPSC amplitude was calculated by averaging the peak amplitudes of all individual NMDAR mEPSCs (amplitude threshold, 7 pA) recorded from a single neuron. To perform kinetic separation of AMPAR and NMDAR mEPSC components, we followed the general procedure described by Watt et al. (2000), except that the specific time points used for measurement of AMPAR and NMDAR mEPSC components were determined based on pharmacologic experiments performed in our cultures. Specifically, AMPA mEPSCs recorded in the presence of AP5 peaked close to 1 ms in both neuronal and mixed cultures, while NMDA mEPSCs recorded in the presence of NBQX, bicuculline, and strychnine peaked at 7.2 ± 0.6 ms in neuronal cultures and at 14.2 ± 1.0 ms in mixed cultures (*n* = 50–60). The residual AMPA mEPSCs were less than 1 pA when measured 7 ms after the peak in neuronal cultures or 15 ms after the peak in mixed cultures, indicating that total mEPSCs were not contaminated by AMPAR components around those time points. For kinetic analysis of the NMDAR/AMPAR ratio, we therefore measured the NMDA mEPSC peak (averaged over 1 ms) from total mEPSCs in a window of 6–10 ms after the AMPA mEPSC peak in neuronal cultures and in a window of 13–17 ms after the AMPA mEPSC peak in mixed cultures. To exclude synapses containing only AMPA components, we selected mEPSC events in which the NMDA mEPSC peak was larger than 2 pA.

### Immunofluorescence

Cells were fixed for 30 min on ice in 4% paraformaldehyde in PBS, washed three times in ice-cold PBS, and blocked for 30 min in a blocking buffer (2% goat serum and 0.25% Triton X-100 in PBS). Cells were then incubated overnight at 4°C with primary antibodies [guinea pig polyclonal anti-synaptophysin (Synaptic System, 1:1000) and mouse monoclonal anti-PSD-95 (6G6-1C9, Abcam, 1:300)], washed three times with PBS at room temperature (RT), and then incubated for an hour at RT with secondary antibodies [Alexa-594-conjugated goat anti-guinea pig (Molecular Probes, 1:1000) and Alexa-488-conjugated goat anti-mouse (Molecular Probes, 1:1000)]. Following 5 min RT incubation with DAPI nuclear dye, coverslips were washed three times in PBS and mounted in Fluoromount-G (Cell Lab, Beckman Coulter).

### Imaging

Images were acquired with a spinning disk confocal microscope (Yokogawa). A healthy-appearing neuron that was about two cell diameters away from its neighbors was placed in the center of the camera field to capture digital images of fluorescence emissions at 488 nm and 594 nm using MetaMorph image capture software. The selected cell was imaged in serial optical sections at 0.33 μm intervals over a total depth of 5 μm, for a total of 15 optical sections. Maximum intensity projections (MIPs) were generated from these sections, yielding 5 MIPs representing 1 μm of depth each; fluorescence intensity in each MIP was quantified as described previously (Ippolito and Eroglu, 2010). Synaptic puncta, defined by co-localization of synaptophysin and PSD-95 labeling, were quantified in the selected regions of interest (ROI; 89 × 89 μm square) using Puncta Analyzer program written by Bary Wark for ImageJ 1.26.^1^ A total of 9 cells from 3 coverslips (each coverslip from a different culture batch) were analyzed for each experimental condition.

### Western Blotting

Cells were lysed on ice with modified RIPA buffer (0.5% SOD, 0.1% SDS, 1% Triton X-100, 150 mM NaCl, 50 mM TrisCl, 1 mM EDTA) supplemented with a protease inhibitor cocktail (Roche). Crude lysates were cleared by centrifugation at 6000 rpm for 10 min at 4°C, solubilized with LDS sample buffer (Invitrogen) supplemented with TCEP reducing reagent (Pierce; final concentration 12.5 mM), and heated for 10 min at 70°C prior to loading; given different cellular compositions of the cultures, samples were loaded on an equal volume basis. Samples were electrophoretically resolved with 4–12% Bis-Tris NuPAGE gels (Invitrogen) and electroblotted to nitrocellulose membranes. Membranes were blocked for 1 h at RT in TBS (150 mM NaCl, 20 mM TrisCl; pH = 7.4) containing 3% nonfat dried milk, incubated with primary antibodies for 2–3 h at RT or overnight at 4°C, washed 4 times at RT, incubated with the corresponding secondary antibody for 1 h at RT, and washed for at least 30 min at RT. Following a final wash in TBS with 0.1% Tween for 10 min at RT, protein-antibody complexes were detected using an ECL chemiluminescent kit (Pierce Biotechnology) and CL-XPosure Film (Thermo Scientific) with a Konica SRX-101A film developer. In a subset of experiments (Figure 10), the Odyssey Fc infrared imaging system (LI-COR Biosciences) was used for detection of the protein-antibody complexes. In these experiments, electrophoresis was performed using 3–8% Tris-Acetate NuPAGE gels (Invitrogen). The membranes were dried at RT for at least 1 h and then blocked with Odyssey Blocking Buffer (LI-COR Biosciences) for 1 h at RT. Odyssey Blocking Buffer supplemented with 0.1% Tween 20 was used for dilution of primary and secondary antibodies, while PBS supplemented with 0.1% Tween 20 was used for washing. Membranes were incubated with primary antibodies for 2 h at RT or overnight at 4°C, washed 4 times (5 min/wash) at RT, incubated with secondary antibodies for 1 h at RT, washed 4 times (5 min/wash) at RT, and then imaged using Odyssey Fc (LI-COR Biosciences) at 700 nm and 800 nm; images were analyzed using Image Studio Imaging Software (LI-COR Biosciences). Primary antibodies included: mouse monoclonal anti-NR1 (BD Biosciences, 1:1000), rabbit monoclonal anti-GluN2A (Millipore, 1:1000), mouse monoclonal anti-GluN2B (BD Biosciences, 1:100 [film] or 1:500 [Odyssey Fc]), rabbit polyclonal anti-phospho-GluN2B (Ser1303) (Millipore, 1:1000), mouse monoclonal anti-GluR1-NT (Millipore, 1:500), mouse monoclonal anti-PSD-95 (NeuroMab, 1:1000), rabbit polyclonal anti-synaptophysin (Santa Cruz Biotechnology, 1:1000) and mouse monoclonal anti-actin (Sigma, 1:5000). Secondary antibodies included goat horseradish peroxidase (HRP)-conjugated anti-rabbit secondary H + L IgG antibody and goat HRP-conjugated anti-mouse secondary H + L IgG antibody (Jackson ImmunoResearch, 1:5000 for both) or goat IRDye® 800CW-conjugated anti-rabbit secondary H + L IgG antibody and goat IRDye® 680RD-conjugated anti-mouse secondary H + L IgG antibody (LI-COR Biosciences, 1:15000 for both).

### Surface Protein Biotinylation

After 2 washes with ice-cold PBS, live cultures were incubated with 1 mg/mL sulfo-NHS-S-S-Biotin (Pierce) for 20 min on ice. Following 2 washes with ice-cold PBS and 2 washes with ice-cold TBS (to remove and quench unbound reagent, respectively), cells were lysed in a modified RIPA buffer. Crude lysates were cleared by centrifugation at 6000 rpm for 10 min at 4°C; 10% of cleared lysate was reserved, while the rest was incubated with 50% NeutraAvidin agarose beads (Pierce) for 2–3 h or overnight at 4°C. After 5 washes with RIPA buffer, bound proteins were eluted with DTT- and TCEP-containing LDS sample buffer by heating for 30 min at 75°C; cleared whole cell lysate (input) and biotinylated protein fractions were then analyzed by Western blotting, as described above.

### Data Fitting and Statistical Methods

Using a nonlinear least-squares algorithm, the dose-response data were fitted to Eq. 1 (where Top and Bottom are maximal and minimal current density values respectively, *y* is the current density obtained in response to a concentration of NMDA *x*, and EC_50_ is the concentration of NMDA that yields the half-maximal current density).

(1) $$y=Bottom+\frac{Top-Bottom}{1+10^{LogEC_{50}-x}}$$

The standard slope (Hill slope = 1) was adopted because (i) estimated Hill coefficients obtained from all groups were similar to 1 [0.9–1.4, as reported previously (Verdoorn and Dingledine, 1988; Patneau and Mayer, 1990; Linsenbardt et al., 2013)]; and (ii) curves were fitted better without a slope parameter.

To determine weighted mean unitary conductance, MATLAB (MathWorks) was used to perform fluctuation analysis on the slowly decaying whole-cell currents evoked by 5 μM NMDA in the presence of 1 μM glycine. (To achieve the sufficiently slow current decay, the wash in these experiments was performed through a whole bath solution exchange rather than through the fast perfusion system used in other experiments.) The mean current and noise variance were calculated from adjacent 500 ms intervals during the portion of the current decay phase that was well fitted with a single exponential; to minimize the effect of changing current amplitude, the current variance was determined after subtracting this single exponential fit from the raw current trace. The mean current-variance plots showed a linear relationship, indicating that only a small fraction of the available channels were open; the weighted mean unitary current was determined from the slope of the linear fit (Watt et al., 2000). The weighted mean single channel conductance was obtained by dividing the mean unitary current by the driving force (−68.8 mV).

Statistical analyses were performed with GraphPad Prism statistical software; the specific test used for each experiment was chosen based on the experimental design and is specified in the main text and figure legends. The data are shown as mean ± SEM, unless indicated otherwise; *p* < 0.05 was considered statistically significant.

## Results

### In Hippocampal Cultures with Mature Synapses, Astrocytes Increase Postsynaptic NMDAR Current Density Through Secretion of a Soluble Factor (or Factors)

**Figure 1:**
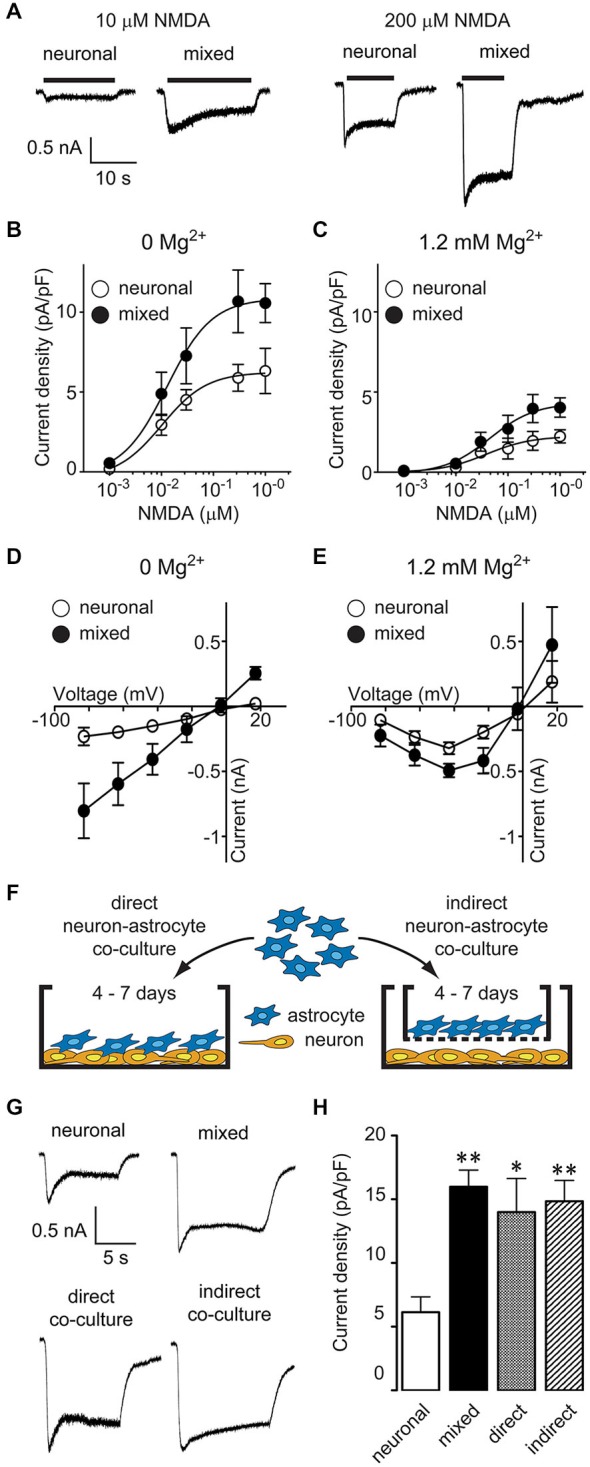
**Astrocyte-secreted factor(s) increase neuronal NMDA-evoked currents in a Mg^2+^- and voltage-independent manner. (A)** Representative current traces evoked by 10 and 200 μM NMDA in the presence of 1 μM glycine in neuronal and mixed cultures (0 Mg^2+^ external solution). **(B,C)** NMDA dose-response curves showed an increase in the maximal NMDA current density, both in the absence **(B)** and presence **(C)** of extracellular Mg^2+^. The plateau current amplitudes were obtained by averaging current values during 1 s at the end of NMDA application; each data point is an average obtained from 2–15 neurons. **(D,E)** Current-voltage relationship of steady state (plateau) NMDA-evoked currents recorded in the absence **(D)** or presence **(E)** of extracellular Mg^2+^. Each data point is an average obtained from 3–8 neurons. **(F)** A schematic diagram of the two neuron-astrocyte co-culture arrangements used for experiments. In direct co-cultures (left), astrocytes were plated directly onto AraC-treated neuronal cultures. In indirect co-cultures, astrocytes were plated on culture inserts that were then placed into wells with AraC-treated neuronal cultures; the insert membrane (1 μm pore size) allowed free exchange of soluble factors but prevented a direct contact between the two cell types. **(G)** Representative current traces evoked by 100 μM NMDA in the presence of 1 μM glycine in neuronal cultures, mixed cultures, direct neuron-astrocyte co-cultures, and indirect neuron-astrocyte co-cultures. **(H)** The average NMDA-evoked current density was significantly larger in mixed cultures and astrocyte co-cultures compared to neuronal cultures (*p < 0.05, **p < 0.01; one-way ANOVA with Dunnett post-test).

To investigate the effect of glia on neuronal NMDARs, we compared NMDAR activity between mixed and predominantly neuronal primary hippocampal cultures by recording whole-cell currents evoked by bath application of NMDA in the presence of glycine, with or without extracellular Mg^2+^. [In our culture system, the neuron-glia ratio is approximately 50%–50% in mixed cultures and 95%–5% in AraC-treated, neuron-enriched cultures; for images of each culture condition, see Habas et al. (2013)]. NMDA-evoked currents were larger in mixed than in neuronal cultures; representative traces from recordings in 0 mM Mg^2+^ are shown in Figure 1A. To examine NMDA current differences in a systematic way, we constructed dose-response curves (Eq. 1) for steady-state current density (plateau current normalized by cell capacitance; Figures 1B,C). NMDA EC_50_ values did not significantly differ between the two culture conditions (0 Mg^2+^: 13 μM in mixed vs. 10 μM in neuronal cultures; 1.2 mM Mg^2+^: 44 μM in mixed vs. 39 μM in neuronal cultures; *p* > 0.05, *F*-test) and were comparable to values previously reported in the literature [7–16 μM in Mg^2+^-free and 25–40 μM in Mg^2+^-containing recording solutions (Verdoorn and Dingledine, 1988; Patneau and Mayer, 1990; Dildy-Mayfield and Leslie, 1991; Jaekel et al., 2006; Linsenbardt et al., 2013)]. In contrast, the maximal NMDAR current density was two-fold greater in mixed than neuronal cultures regardless of the Mg^2+^ concentration used (0 Mg^2+^: 10.6 ± 1.2 pA/pF vs. 6.3 ± 1.4 pA/pF; 1.2 mM Mg^2+^: 4.0 ± 0.6 pA/pF vs. 2.2 ± 0.4 pA/pF, *p* < 0.05, *F*-test). Since the voltage dependence of Mg^2+^ block shapes the voltage dependence of NMDAR currents (Nowak et al., 1984), this finding suggested that glial modulation of NMDARs was voltage independent; to investigate this further, we directly assessed the current-voltage (I-V) relationship of NMDA-evoked currents (Figures 1D,E). In Mg^2+^-free recording solution (Figure 1D), I-V curves were linear with a reversal potential of ~0 mV in both culture conditions, as described previously (Nowak et al., 1984); however, the current amplitudes were significantly greater in mixed than neuronal cultures (*p* < 0.0001; two-way ANOVA). In the presence of 1.2 mM Mg^2+^ (Figure 1E), I-V curves exhibited a typical block at negative voltages while maintaining the significant amplitude difference between the two culture conditions (*p* < 0.05; two-way ANOVA).

Mixed hippocampal cultures used in these experiments contain a heterogeneous population of glia. To determine whether the increase in neuronal NMDAR current density was mediated by astrocyte-secreted factor(s), we used two different modes of neuron-astrocyte co-culture, as described previously (Habas et al., 2013): in direct co-cultures, there was a physical contact between neurons and astrocytes, while in indirect co-cultures, the two cell types shared the same medium but were not in a direct contact (Figure 1F). The density of NMDAR currents evoked by 100 μM NMDA and 1 μM glycine (in the absence of Mg^2+^) was comparable between mixed cultures (16.0 ± 1.3 pA/pF), direct neuron-astrocyte co-cultures (14.0 ± 2.6 pA/pF), and indirect neuron-astrocyte co-cultures (14.8 ± 1.6 pA/pF) and was approximately twice as large as the density of NMDAR currents in neuronal cultures (6.1 ± 1.2 pA/pF) (Figures 1G,H; one-way ANOVA with *post hoc* Dunnett test, *p* < 0.01, *p* < 0.05, and *p* < 0.01, respectively; *n* = 8). Taken together, these results demonstrate that astrocytes increase neuronal NMDAR current density in a voltage-independent fashion through a secretion of a soluble factor (or factors).

### In the Presence of Astrocytes, there is an Increase in the Size of NMDAR-Mediated Synaptic Currents but No Significant Change in the Overall Synaptic Density

**Figure 2:**
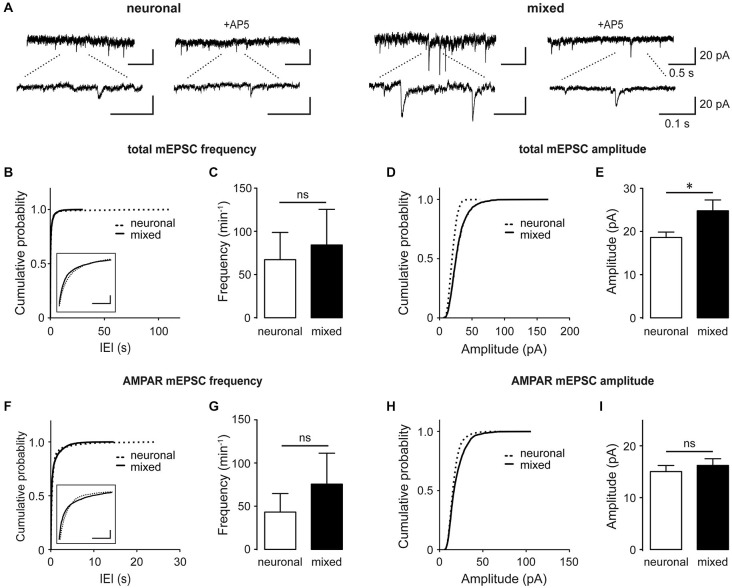
**Culturing neurons with glia increases the total mEPSC amplitude without significantly affecting AMPAR mEPSCs. (A)** Spontaneous mEPSCs recorded at −60 mV in neuronal and mixed cultures. Total mEPSCs (left) contain both AMPAR and NMDAR-mediated components; AMPAR-mediated mEPSCs were isolated using the NMDAR blocker AP5 (100 μM, right). Bottom traces are an expanded view of top traces. **(B–E)** Cumulative probability and mean plots of total mEPSC frequency **(B,C)** and peak amplitude **(D,E). (F–I)** Cumulative probability and mean plots of AMPAR mEPSC frequency **(F,G)** and peak amplitude **(H,I)**. For **(B–I)**, cumulative probability plots were generated using all events from 7 cells for each culture condition; summary bar graphs show values averaged from 7 cells for each group. (*p < 0.05, *t*-test). In **(B)** and **(F)**, the segments of cumulative probability curves with the largest difference between neuronal and mixed cultures are shown in insets (horizontal scale bars, 1 s; vertical scale bars, cumulative probability of 0.1); in both plots, the differences between the two curves were statistically significant but very small and thus likely not biologically significant.

Several studies have shown that astrocyte-secreted factors regulate synapse formation and function during development of retinal ganglion cells (Pfrieger and Barres, 1997; Ullian et al., 2001; Christopherson et al., 2005; Allen et al., 2012), raising the possibility that the astrocyte-induced increase in the whole-cell NMDAR current density was a result of the increase in the overall synapse density. However, glial influence on excitatory synapses varies with neuronal cell type (Steinmetz et al., 2006) and developmental stage (Xu et al., 2010). To directly examine glial effect(s) on excitatory synapses in our hippocampal culture system, we recorded spontaneous synaptic activity at DIV13–17, when synaptogenesis is known to reach a plateau (Grabrucker et al., 2009; Xu et al., 2010). Total miniature excitatory postsynaptic excitatory currents (mEPSCs), which contain both AMPAR- and NMDAR-mediated components, were recorded in the presence of 1 μM tetrodotoxin (TTX) to block action potentials and 20 μM bicuculline to block inhibitory synaptic transmission (Figures 2A–E). Cumulative probability plots of the mEPSC inter-event-interval (IEI; Figure 2B) and amplitude (Figure 2D) showed small but statistically significant changes in the overall event distribution (total mEPSC IEI: *p* < 0.005, total mEPSC amplitude: *p* < 0.001, Kolmogorov-Smirnov [KS] test); however, the mean mEPSC frequency did not significantly differ between the two culture conditions (84.3 ± 41.2 min^−1^ for mixed vs. 67.2 ± 31.6 min^−1^ for neuronal cultures, *n* = 7, *p* = 0.6, *t*-test; Figure 2C). In contrast, the peak total mEPSC amplitude was 33% larger in mixed than neuronal cultures (24.8 ± 2.5 pA vs. 18.6 ± 1.3 pA respectively, *n* = 7, *p* < 0.05, *t*-test; Figure 2E). When recordings were performed in the presence of 100 μM AP5 to block the NMDAR mEPSC component (Figures 2A,F–I), we again observed very small but statistically significant changes in the distribution of AMPAR mEPSC IEI (Figure 2F) and amplitude (Figure 2H) (*p* < 0.001, KS test). However, there was no statistical significance in the mean frequency (76.4 ± 35.6 min^−1^ in mixed vs. 43.2 ± 22.0 min^−1^ in neuronal cultures, *n* = 7, *p* > 0.05, *t*-test; Figure 2G) or the mean peak amplitude of AMPAR mEPSCs between the two culture conditions (16.2 ± 1.3 pA in mixed vs. 15.0 ± 1.2 pA in neuronal cultures; *n* = 7, *p* > 0.05, *t*-test; Figure 2I).

**Figure 3:**
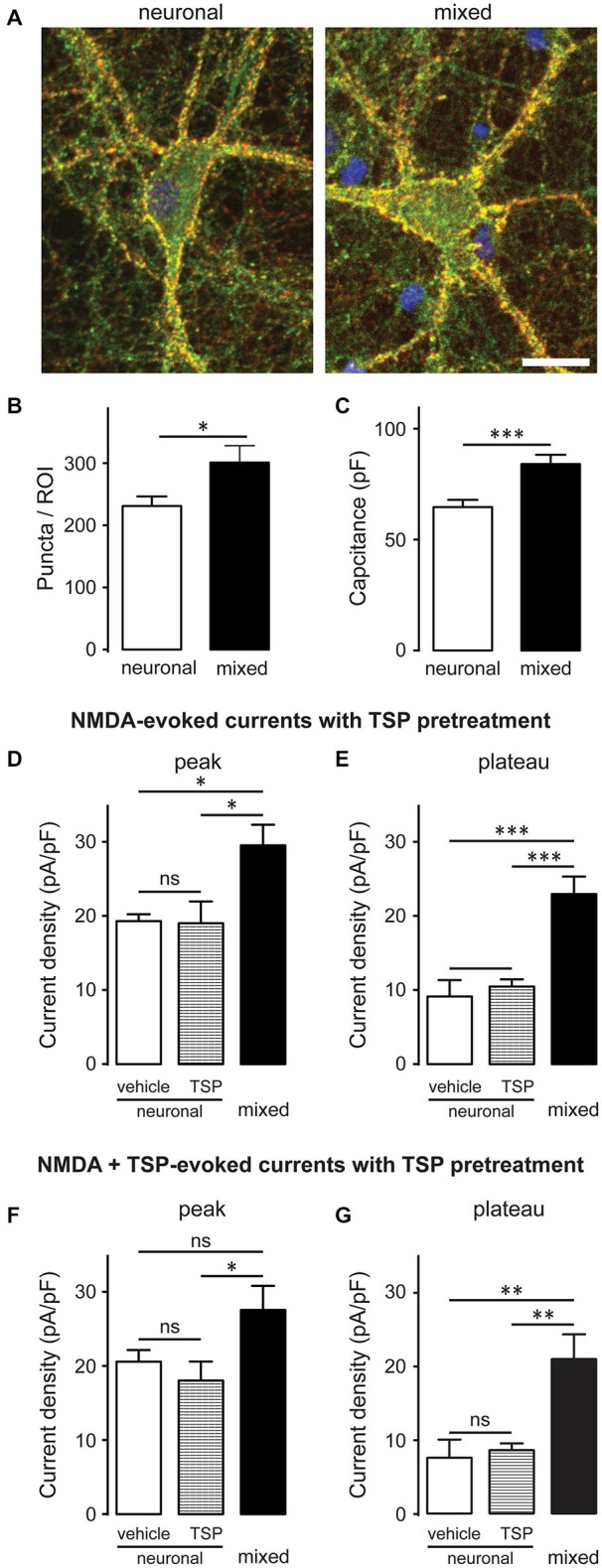
**Glia-induced increase in the NMDAR current density is not due to an appreciable change in synaptic density and cannot be mimicked by thrombospondin (TSP) treatment of neuronal cultures. (A)** Synaptic puncta were visualized by co-localization of presynaptic marker synaptophysin (red) and the postsynaptic marker PSD-95 (green). Scale bar, 20 μm. **(B,C)** The number of synaptic puncta per ROI (which included the soma and proximal dendrites of each imaged neuron) was higher in mixed than neuronal cultures **(B)**; however, this increase was proportional to the increase in the neuronal membrane capacitance (a proxy for the neuronal surface area) observed in mixed cultures **(C)**. (**B,C**: *p < 0.05, ***p < 0.001; *t*-test) **(D,E)** 10-day pretreatment of neuronal cultures with 5 μg/ml TSP had no effect on NMDAR current density measured at either peak or plateau time points; for comparison, NMDAR current density in mixed sister cultures was significantly larger than the current density in neuronal cultures after either pretreatment. Control neuronal cultures were pretreated with vehicle (0.07% glycerol); NMDA currents were evoked by application of 200 μM NMDA in the presence of glycine. **(F,G)** Acute TSP treatment (5 μg/ml) had no effect on NMDAR current density in neuronal cultures. In these experiments, neuronal cultures were first pretreated with 5 μg/ml TSP or vehicle, as described for **(D,E)**; TSP or vehicle were then co-applied with NMDA in the presence of glycine. There was no difference in the mean NMDAR current density between TSP- and vehicle-treated cultures at either time point (peak and plateau); as expected, NMDAR current density was significantly larger in mixed sister cultures. (**D–G**: *p < 0.05, **p < 0.01, ***p < 0.001, one-way ANOVA with Bonferroni post-test).

A subset of glutamatergic synapses contains only NMDARs (Hanse et al., 2013), rendering them AMPAR-silent and thus undetectable in standard mEPSC recordings; therefore, we also compared the overall number of synapses between the two culture conditions. Neurons were immunostained with antibodies against synaptophysin (a presynaptic marker; red) and PSD-95 (a postsynaptic marker; green), as described previously (Ullian et al., 2001; Christopherson et al., 2005; Ippolito and Eroglu, 2010); synapses were detected as yellow puncta, indicating co-localization of the two markers (Figure 3A). The number of synaptic puncta per ROI (which included the soma and proximal dendrites of imaged neurons) was 30% greater in mixed than neuronal cultures, a statistically significant difference (301.1 ± 27.2 vs. 231.2 ± 15.4 puncta/ROI; *n* = 9, *p* < 0.05, *t*-test; Figure 3B). However, neuronal surface area (measured as membrane capacitance) was also 30% greater in mixed than neuronal cultures (84.1 ± 4.2 pF vs. 64.7 ± 3.3 pF respectively, *n* = 25, *p* < 0.001, *t*-test; Figure 3C). These measurements were performed on different cell populations and thus could not be used for direct calculation of the number of synapses per unit of the membrane capacitance; nonetheless, together they strongly suggest that synaptic density did not appreciably differ among the two culture conditions. In agreement with this finding, thrombospondin [astrocyte-secreted factor that promotes retinal ganglion cell synaptogenesis (Christopherson et al., 2005)] had no effect on the NMDAR current density in neuronal cultures (Figures 3D–G). Collectively, these data indicate that (i) the presence of glial cells does not have a major effect on the density of excitatory synapses in mature hippocampal cultures; and (ii) that the two-fold difference in whole-cell NMDAR current density between mixed and neuronal cultures cannot be explained by the difference in synaptic density between the two culture conditions.

**Figure 4:**
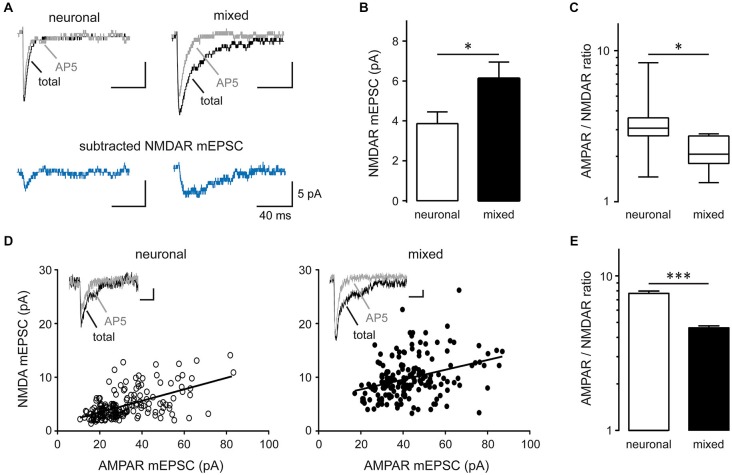
**Synaptic AMPAR/NMDAR ratio is decreased in mixed compared to neuronal cultures. (A)** Representative average mEPSC traces recorded before (total mEPSC) and after AP5 treatment (AMPAR mEPSC); the NMDAR mEPSC trace was obtained by subtracting the average AMPAR mEPSC trace from the average total mEPSC trace. **(B)** The peak NMDAR mEPSC amplitude (obtained from averaged NMDAR mEPSC traces) was significantly larger in mixed than neuronal cultures (*p < 0.05). **(C)** The AMPAR/NMDAR mEPSC ratio (calculated from the peak amplitudes of averaged AMPAR and NMDAR traces) was significantly smaller in mixed cultures. The data were not normally distributed and are shown as box-and-whiskers plots (box edges, 25th and 75th percentiles; whiskers, 10th and 90th percentiles; *p < 0.05, Mann-Whitney nonparametric test on ranks). **(D)** Peak amplitudes of AMPAR and NMDAR mEPSC components obtained from individual total mEPSCs (see Materials and Methods for details) were plotted across neurons (180 events from *n* = 7 for each culture condition); the solid line is the best straight line fit (neuronal, *r*^2^ = 0.32; mixed, *r*^2^ = 0.12). The correlation coefficient decreased from 0.57 in neuronal to 0.34 in mixed cultures, indicating differential regulation of AMPAR and NMDAR mEPSCs by glia. Insets: representative single mEPSC traces before and after AP5. Horizontal scale bars, 10 ms; vertical scale bars, 5 pA. **(E)** The AMPAR/NMDAR mEPSC ratio obtained from analysis of individual mEPSC events was significantly smaller in mixed cultures (***p < 0.001, *t*-test).

**Figure 5:**
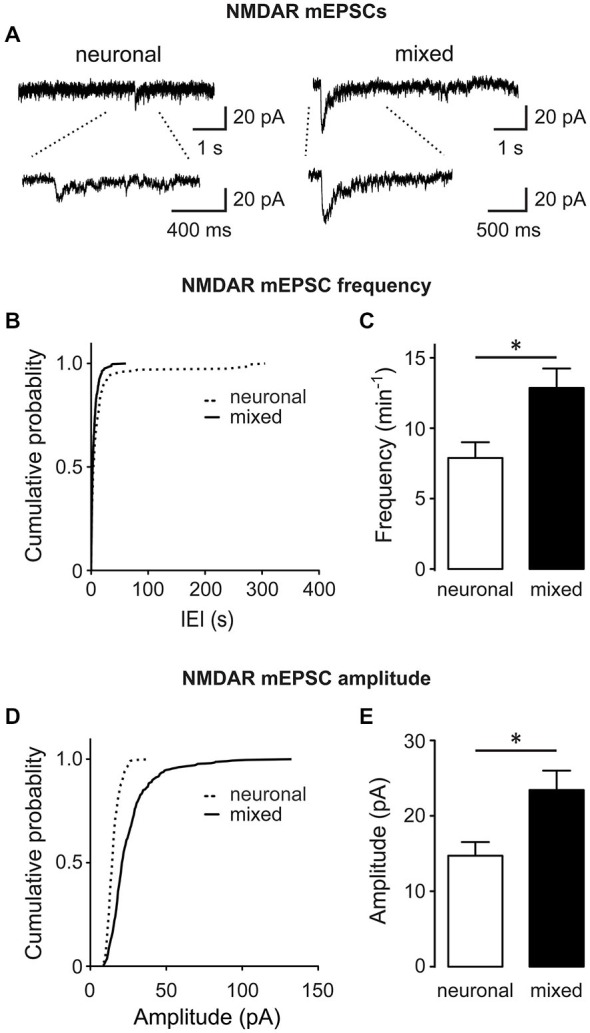
**The frequency and amplitude of directly recorded NMDAR mEPSCs are increased in the presence of glia. (A)** Representative NMDAR mEPSC traces recorded at −60 mV from neuronal and mixed cultures in the presence of TTX, NBQX, bicuculline and strychnine; bottom traces show recordings on an expanded time scale. **(B–E)** Cumulative probability, mean frequency, and mean amplitude plots of NMDAR mEPSCs are shown; both frequency and amplitude of NMDAR mEPSCs were significantly larger in mixed cultures (*p < 0.05, *t*-test). Of note, the peak NMDAR mEPSC amplitudes obtained from this analysis were larger than the peak NMDAR mEPSC amplitudes obtained by subtracting the average AMPAR mEPSC traces from the average total mEPSC traces (Figure 4B); the most likely explanation for this discrepancy is our inability to resolve small and medium-sized (<7 pA) NMDAR mEPSC events from the background noise in direct recordings, biasing the overall analysis to the largest mEPSCs.

The mean amplitude of total mEPSCs (Figure 2E), but not AMPAR mEPSCs (Figure 2I), was significantly larger in mixed than neuronal cultures, suggesting an increase in the NMDAR-mediated mEPSC component. Indeed, the mean amplitude of average NMDAR mEPSCs (obtained by subtraction of the average AMPAR mEPSC trace from the average total mEPSC trace for each neuron; Figure 4A) was significantly larger in mixed than neuronal cultures (6.1 ± 0.8 vs. 3.9 ± 0.6 pA, respectively; *n* = 7, *p* < 0.05, *t*-test; Figure 4B), resulting in a decreased AMPAR/NMDAR ratio in mixed cultures (median of 2.1 in mixed vs. 3.1 in neuronal cultures, *n* = 7, *p* < 0.05, Mann-Whitney test; Figure 4C). To determine whether this increase in the average NMDAR mEPSC reflects a population of new synapses or an increase in the NMDAR mEPSC component of existing synapses, we examined the AMPAR/NMDAR ratio at individual synapses using kinetics analysis that is based on distinctive inactivation properties of these two types of glutamate receptors, as described previously (Edmonds et al., 1995; Watt et al., 2000). When NMDAR and AMPAR mEPSC amplitudes from each event were plotted against each other, the correlation coefficient became smaller in mixed cultures (0.34) than in neuronal cultures (0.57) (Figure 4D), indicating that AMPAR and NMDAR components of mEPSC were not proportionally scaled at individual synapses. In agreement with this observation and with the results from the average mEPSC analysis (Figures 4A–C), the AMPAR/NMDAR ratio obtained from individual mEPSCs was significantly smaller in mixed than neuronal cultures (4.6 ± 0.1 vs. 7.7 ± 0.3, *n* = 7; *p* < 0.001, *t*-test; Figure 4E). To directly measure differences in NMDAR mEPSCs between mixed and neuronal cultures, we performed recordings in the presence of the AMPAR blocker NBQX (10 μM) in addition to TTX, bicuculline, and strychnine (1 μM) (Figure 5A). Cumulative plots of the NMDAR mEPSC IEI and amplitude showed a large and statistically significant change in the overall event distribution (total mEPSC IEI: *p* < 0.005, total mEPSC amplitude: *p* < 0.001, KS test; Figures 5B,D). In addition, there was a significant increase in the NMDAR mEPSC frequency and peak amplitude in mixed compared to neuronal cultures (12.9 ± 1.4 vs. 7.9 ± 1.1 min^−1^ and 23.4 ± 2.6 vs. 14.0 ± 1.4 pA, *n* = 6–7, *p* < 0.05, *t*-test; Figures 5C,E). Taken together, these results demonstrate that (i) NMDAR-mediated synaptic currents in mature hippocampal neurons are potentiated in the presence of astrocytes and (ii) astrocytes differentially affect NMDARs and AMPARs at individual synapses, resulting in a lower AMPAR/NMDAR ratio in mixed cultures.

### Astrocytes Selectively Regulate Synaptic GluN2B NMDARs

**Figure 6:**
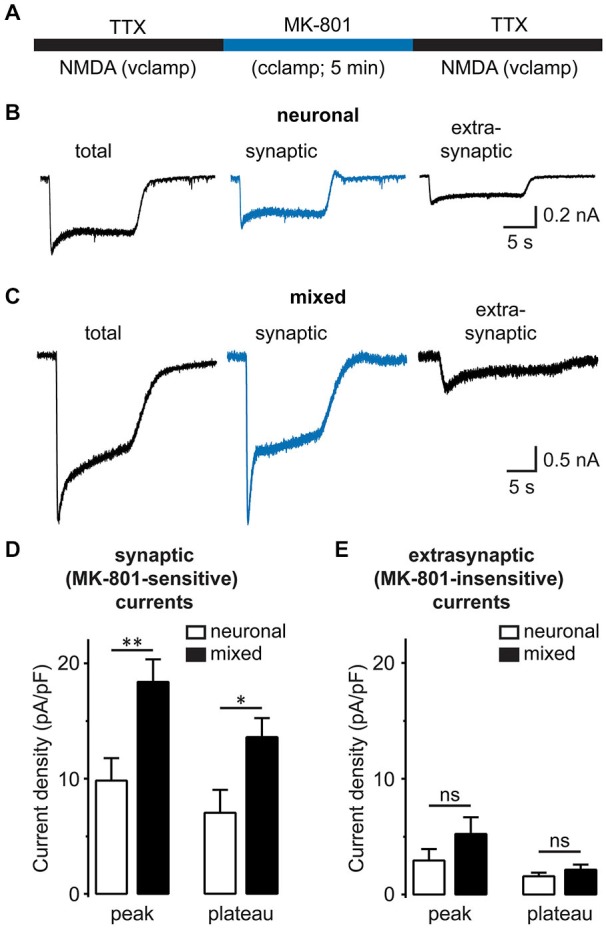
**Synaptic, but not extrasynaptic, NMDA-evoked currents are increased in the presence of glia. (A)** A schematic diagram of the drug application protocol used to separate synaptic from extrasynaptic NMDAR currents. The total whole-cell NMDAR currents were first recorded in a Mg^2+^-free external solution containing TTX. To block synaptic NMDA currents, neurons were then switched for 5 min to the current-clamp configuration and a TTX-free solution containing Mg^2+^ and 10 μM MK-801; under these conditions, the spontaneous neuronal firing activates synaptic (but not extrasynaptic) NMDAR channels, which are then irreversibly blocked by the activity-dependent, irreversible NMDAR channel blocker MK-801. Finally, neurons were switched back to the regular recording solution and voltage clamp configuration to record the residual extrasynaptic NMDA currents. Between patch-clamp recording configuration switches, neurons were perfused for 2 min.** (B,C)** Representative current traces evoked by 200 μM NMDA in the presence of glycine in neuronal **(B)** and mixed cultures **(C)**. Total NMDAR currents (left) were recorded before MK-801 treatment, while residual/extrasynaptic NMDAR currents (right) were recorded after MK-801 treatment, as described in **(A)**. Synaptic NMDA currents (center) were obtained by subtracting MK-801-insensitive from total NMDA currents. **(D,E)** Mean peak and steady-state (plateau) current densities are plotted for synaptic **(D)** and extrasynaptic NMDA currents **(E)**. The peak current amplitude (averaged over 5 ms) was determined within 1 s of NMDA application; the plateau current amplitude was obtained by averaging during the last 1 s of 10–15 s NMDA application. Both peak and plateau synaptic NMDAR currents were greater in mixed cultures, but extrasynaptic currents did not significantly differ between the two culture conditions (*p < 0.05, **p < 0.01, *t*-test).

Synaptic and extrasynaptic NMDARs have a similar subunit composition, but different cellular functions (Hardingham and Bading, 2010). To determine whether glia differentially modulate these two NMDAR populations, we used the activity-dependent open NMDAR channel blocker MK-801 to isolate synaptic from extrasynaptic NMDAR currents, as described previously (Papadia et al., 2008): after synaptic NMDA currents were irreversibly blocked by MK-801 application during a period of spontaneous neuronal activity, residual extrasynaptic NMDAR currents were evoked by bath application of NMDA (Figure 6A; representative recordings from neuronal and mixed cultures are shown in Figures 6B,C). In agreement with the data from our mEPSC experiments, the density of the synaptic (MK-801-sensitive) NMDAR current was significantly greater in mixed cultures at both peak and plateau time points [peak: 18.4 ± 2.0 vs. 9.8 ± 1.9 pA/pF, *p* < 0.01; plateau: 13.5 ± 1.6 vs. 7.0 ± 2.0 pA/pF, *p* < 0.05; *n* = 12–13, *t*-test (Figure 6D)]. In contrast, the density of the extrasynaptic (MK-801-insensitive) NMDAR current did not significantly differ between the two culture conditions [peak: 5.2 ± 1.4 pA/pF in mixed vs. 2.9 ± 1.0 pA/pF in neuronal cultures, *p* > 0.05; plateau: 2.1 ± 0.4 in mixed vs. 1.6 ± 0.3 pA/pF in neuronal cultures, *p* > 0.05; *n* = 12–13, *t*-test (Figure 6E)].

**Figure 7:**
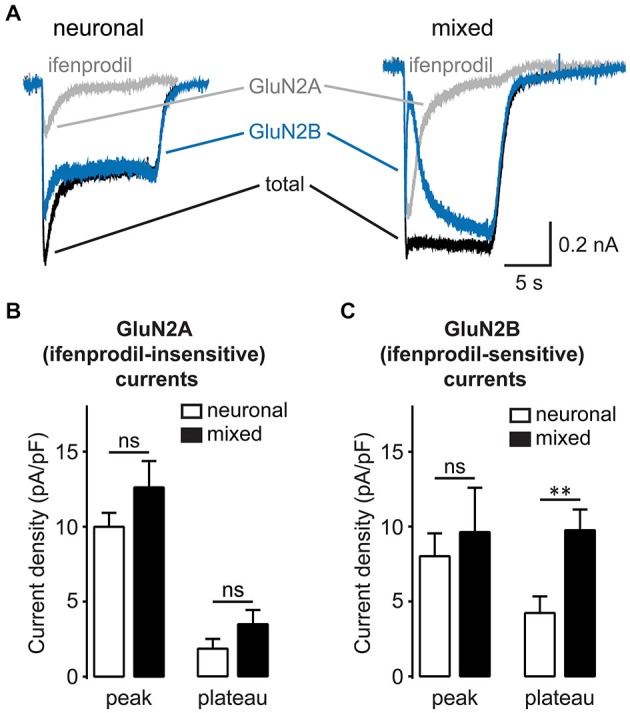
**Glia-induced enhancement of NMDA-evoked currents is GluN2B subunit specific. (A)** Representative current traces evoked by 200 μM NMDA in the presence of glycine recorded before and after treatment with the GluN2B-specific antagonist ifenprodil (3 μM). Ifenprodil-sensitive GluN2B NMDAR currents (blue traces) were obtained by subtracting the ifenprodil-insensitive GluN2A currents (gray traces) from the total NMDAR currents (black traces). **(B,C)** Mean peak and steady-state (plateau) current densities are plotted for GluN2A **(B)** and GluN2B currents **(C)**. The plateau, but not peak, GluN2B currents were significantly larger in mixed cultures, while GluN2A currents did not significantly differ between the two culture conditions at either time point (**p < 0.01, *t*-test).

A significant fraction of the synaptic NMDAR current is carried by channels that incorporate slowly-inactivating GluN2B subunit, although these channels are found at both subcellular locations (Harris and Pettit, 2007). To examine the subunit specificity of glial NMDAR potentiation, we recorded NMDA-evoked currents in the presence of ifenprodil (3 μM), a GluN2B-selective inhibitor (Williams, 1993; Figure 7A). The density of ifenprodil-insensitive, largely GluN2A-mediated NMDAR currents was not significantly different between the two culture conditions [peak: 12.6 ± 1.8 pA/pF in mixed vs. 10.0 ± 0.9 pA/pF in neuronal cultures, *p* > 0.05; plateau: 3.5 ± 1.0 pA/pF in mixed vs. 1.8 ± 0.7 pA/pF in neuronal cultures, *p* > 0.05; *n* = 8–10, *t*-test (Figure 7B)]. In contrast, ifenprodil-sensitive, GluN2B-mediated currents were significantly larger in mixed cultures when measured at the plateau, but not at the peak time point [peak: 9.6 ± 3.0 vs. 8.0 ± 1.5 pA/pF, *p* > 0.05; plateau: 9.8 ± 1.4 vs. 4.2 ± 1.1 pA/pF, *p* < 0.001; *n* = 8–10, *t*-test (Figure 7C)]. Taken together, these data indicate that synaptic GluN2B-containing NMDAR channels are the target of glial modulation.

### Astrocytes Regulate NMDAR Channel Activity Through a PKC-Mediated Mechanism

**Figure 8:**
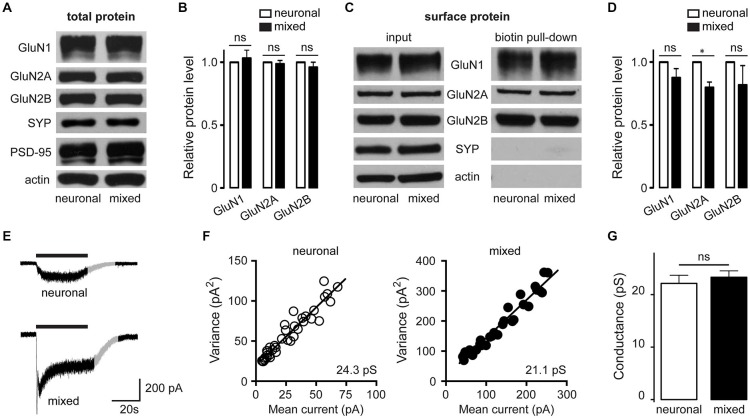
**In the presence of glia, there is no increase in the neuronal expression of NMDAR channel subunits or the size of the unitary NMDAR channel conductance. (A,B)** Expression of GluN1, GluN2A and GluN2B proteins in the whole cell lysates from neuronal and mixed cultures; a representative Western blot **(A)** and quantification of data from 3 independent experiments **(B)** are shown. Synaptophysin (SYP), PSD-95, and actin were used as loading controls; for quantification, NMDAR subunit levels were normalized to SYP level. **(C,D)** Expression of biotinylated (surface-expressed) GluN1, GluN2A and GluN2B proteins in neuronal and mixed cultures; a representative Western blot **(C)** and quantification of data from 3 independent experiments **(D)** are shown. Note that intracellular proteins (SYP and actin) are not detectable in this preparation; for quantification, NMDAR subunit expression was normalized to SYP levels in the input (whole cell lysate) portion of the same samples. In the presence of glia, there was a small but statistically significant decrease in the GluN2A subunit surface expression, while the GluN2B subunit surface expression did not differ between the two culture conditions (*p < 0.05, *t*-test). **(E-G)** Fluctuation analysis was used to determine the weighted mean unitary NMDAR channel conductance for each culture condition. **(E)** Representative recordings (evoked by application of 5 μM NMDA in the presence of 1 μM glycine); the gray portion of each trace highlights the section used for fluctuation analysis **(F)** Mean current-variance plots from the two traces showed in **(E)**; weighted mean unitary conductance values were derived from the slope of the linear fit. **(G)** The weighted mean unitary NMDAR channel conductance did not significantly differ between the two culture conditions (*p* > 0.05, *t*-test).

Increased whole-cell current density can be due to an increase in the number of ion channels on the cell surface or to an increase in the activity of ion channels already localized to the plasma membrane. To differentiate between these two possibilities, we examined the total and cell surface expression of the major hippocampal NMDAR subunits, GluN1, GluN2A and GluN2B. In contrast to glial modulation of AMPAR [which involves increased cell surface expression and clustering of AMPAR GluA1 subunits (Allen et al., 2012)], neither total protein level nor cell surface expression of GluN1, GluN2A and GluN2B subunits was significantly higher in mixed compared to neuronal cultures (Figures 8A–D), indicating that the increase in the whole-cell current density was due to a change in the activity of the surface-expressed NMDAR channels. Ion channel activity is a function of two different aspects of single channel behavior: single channel (unitary) conductance and single channel kinetics. While evaluation of single channel kinetics requires patch recordings not suitable for analysis of physically inaccessible synaptic channels (Traynelis and Jaramillo, 1998), unitary conductance can be determined from fluctuation analysis of macroscopic currents (Sigworth, 1981). To further elucidate the mechanism mediating astrocytic enhancement of NMDAR channel activity, we therefore performed fluctuation analysis of the slowly decaying whole-cell currents evoked by 5 μM NMDA (Mayer et al., 1988; Watt et al., 2000). Figures 8E,F show representative current traces and mean current-variance plots for one neuron from each culture condition, with the slope of the linear fit yielding the weighted mean unitary conductances of 24.3 pS (neuron without glia) and 21.1 pS (neuron with glia) for the two neurons shown; these values are smaller than the NMDAR conductance values obtained from outside-out patch recordings [40–50 pS (Jahr and Stevens, 1987; Ascher et al., 1988; Robinson et al., 1991)], but are in line with a broad range of NMDAR single channel conductance values obtained from fluctuation analysis [9–41 pS (Ascher et al., 1988; Sah et al., 1989; Watt et al., 2000)]. Importantly, the mean weighted unitary NMDAR channel conductance did not significantly differ between the two culture conditions (22.2 ± 1.5 pS in neuronal vs. 23.3 ± 1.2 pS in mixed cultures, *n* = 6–7, *p* > 0.05, *t*-test; Figure 8G), suggesting that a change in the single channel kinetics rather than a change in the single channel conductance is the key biophysical mechanism mediating the astrocytic modulation of synaptic GluN2B NMDAR channels.

**Figure 9:**
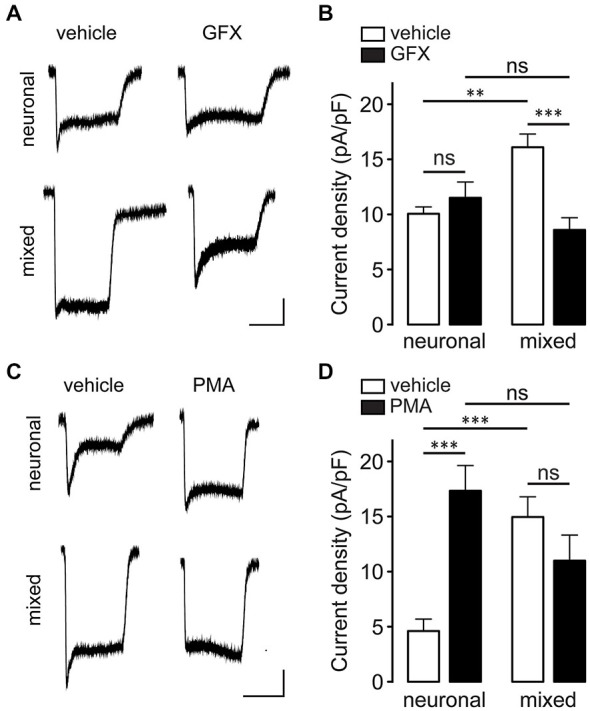
**Glia-induced NMDAR current enhancement is mediated by PKC. (A)** Representative current traces evoked by 100 μM NMDA in the presence of 1 μM glycine recorded after 40 min pretreatment with either vehicle (0.1% DMSO) or GFX (5 μM). Horizontal scale bar, 10 s; vertical scale bar, 250 pA. **(B)** GFX pretreatment blocked the glia-induced potentiation of NMDA-evoked currents in mixed cultures but had no effect in neuronal cultures (**p < 0.01, ***p < 0.001, two-way ANOVA with Bonferroni post-test). **(C)** Representative current traces evoked by 100 μM NMDA in the presence of 1 μM glycine recorded after 10 min pretreatment with either vehicle (0.1% DMSO) or PMA (100 nM). Horizontal scale bar, 10 s; vertical scale bar, 250 pA. **(D)** PMA pretreatment significantly increased NMDAR current density in neuronal cultures but had no effect in mixed cultures (***p < 0.001, two-way ANOVA with Bonferroni post-test).

**Figure 10:**
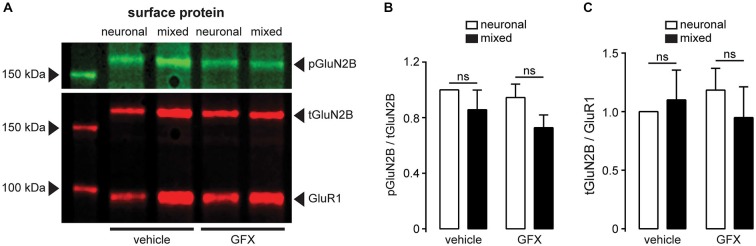
**The degree of GluN2B-Ser1303 phosphorylation is not affected by the presence of glia or PKC inhibition. (A)** A representative immunoblot of surface-expressed proteins showing GluN2B (tGluN2B), phospho-GluN2B-Ser1303 (pGluN2B), and AMPAR subunit GluR1 (loading control); cultures were treated for 3.5 h with either vehicle (0.1% DMSO) or GFX (5 μM). **(B)** Quantification of pGluN2B surface expression normalized to tGluN2B; neither the presence of glia nor the inhibition of PKC affected the phosphorylation status of Ser1303 in the C terminus of GluN2B (*n* = 6; two-way ANOVA with Sidak post-test). **(C)** Quantification of tGluN2B surface expression normalized to GluR1. In agreement with the data shown in Figure 8, neither the culture condition nor the PKC inhibition affected the cell surface expression of GluN2B subunit (*n* = 6; two-way ANOVA with Sidak post-test).

PKC was previously shown to increase the whole-cell NMDAR current by phosphorylation of Ser1303 and/or Ser1323 in the C-terminus of GluN2B (Liao et al., 2001). To investigate whether PKC is involved in the astrocyte-induced potentiation of NMDAR currents, neuronal and mixed cultures were pretreated with PKC inhibitor GF 109203X (GFX; 5 μM) for 40 min prior to recording (Figures 9A,B). [GFX inhibits PKC with IC_50_ of ~10 nM (Toullec et al., 1991), but in physiological experiments it is typically used at concentrations between 1–10 μM.] GFX pretreatment significantly attenuated NMDAR current density in mixed cultures (16.1 ± 1.2 vs. 8.6 ± 1.1 pA/pF; *n* = 6–10, *p* < 0.01, two-way ANOVA with Bonferroni multiple comparison post-test) but had no effect in neuronal cultures (10.1 ± 0.6 vs. 11.5 ± 1.4 pA/pF; *p* > 0.05); as a result, the difference in NMDAR current density between the two culture types, which was significant in vehicle-pretreated cultures (*p* < 0.01), was completely abolished following GFX pretreatment (*p* > 0.05). In agreement with these results, PKC activator phorbol-12-myristate-13-acetate (PMA) had the opposite effect (Figures 9C,D): pretreatment with 100 nM PMA significantly increased NMDAR current density in neuronal cultures (17.4 ± 2.3 vs. 4.6 ± 1.1 pA/pF; *n* = 8–12, *p* < 0.001, two-way ANOVA with Bonferroni multiple comparison post-test) but had no effect in mixed cultures (15.0 ± 1.8 vs. 11.0 ± 2.3 pA/pF; *p* > 0.05); consequently, the difference in NMDAR current density between the two culture types (which was significant in vehicle-pretreated cultures; *p* < 0.001) was not significant following PMA pretreatment (*p* > 0.05). Interestingly, however, there was no significant difference in the phosphorylation status of GluN2B Ser1303 between the two culture conditions in either vehicle- or GFX-treated cultures (Figure 10; phosphorylation of Ser1323 could not be evaluated due to the lack of phospho-specific antibodies targeting that site). Taken together, these data indicate that PKC mediates the astrocyte-induced increase in the synaptic GluN2B channel activity, but that this effect does not involve phosphorylation of GluN2B-Ser1303.

## Discussion

**Figure 11:**
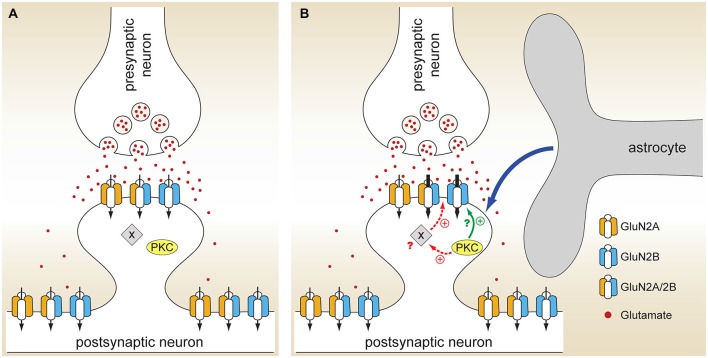
**Model for astrocyte-mediated regulation of neuronal NMDARs. (A)** When hippocampal neurons are cultured in the absence of glia, glutamate released from the presynaptic terminal activates both GluN2A and GluN2B NMDARs localized at synapses. **(B)** In the presence of glia, astrocytes secrete a currently unidentified soluble factor or factors (blue arrow) that activate PKC. Through phosphorylation of either the GluN2B subunit itself (solid green arrow) or, more likely, an ancillary protein (X) associated with the NMDAR signaling complex (dashed red arrows), PKC activation leads to an increase in the activity of synaptic GluN2B NMDARs without affecting the activity of either synaptic GluN2A NMDARs or extrasynaptic GluN2B NMDARs; it remains to be seen whether astrocytic modulation is specific for GluN1/2B heterodimers or it also affects GluN1/2A/2B heterotrimers (as shown).

Neuronal NMDARs play a critical role in synaptic plasticity, neuroprotection, and neurotoxicity, but relatively little is known about their regulation by glial cells. Our study identified a novel form of astrocytic NMDAR modulation that occurs in a subunit- and subcellular localization-dependent manner. The density of NMDA-evoked whole-cell current was approximately doubled in neurons cultured in the presence of a mixed population of glia compared to neurons cultured alone (Figure 1), indicating a change in either expression or function of postsynaptic NMDAR channels. The glial effect was mediated by (an) astrocyte-secreted soluble factor(s) (Figure 1), was Mg^2+^ and voltage independent (Figure 1), and could not be explained by an appreciable change in the synaptic density (Figure 3). Instead, we found that the peak amplitude of total and NMDAR mEPSCs, but not AMPAR mEPSCs, was significantly larger in mixed cultures, resulting in a decreased synaptic AMPAR/NMDAR ratio (Figures 2, 4, 5). Astrocytic modulation was restricted to synaptic NMDARs that contain the GluN2B subunit (Figures 6, 7), did not involve an increase in the cell surface expression of NMDAR subunits (Figure 8), and was mediated by PKC (Figure 9). Collectively, these data show that astrocyte-secreted soluble factor(s) can fine-tune synaptic NMDAR activity through the PKC-mediated regulation of GluN2B NMDAR channels already present at postsynaptic sites, presumably on a rapid time scale (Figure 11).

Based on studies of transgenic mice that express dominant negative SNARE (dnSNARE) construct under the control of a glial fibrillary acidic protein (GFAP) promoter, it was previously suggested that astrocytes regulate the surface expression of GluN2A and GluN2B subunits by increasing the extracellular concentration of adenosine, which activates A1 receptors and leads to tyrosine phosphorylation of Src kinase and the GluN2B subunit (Deng et al., 2011). However, these findings have been called in question by a recent study by Fujita et al., which showed that GFAP-dnSNARE mice (i) express dnSNARE not just in astrocytes but also in a subset of cortical and hippocampal neurons and (ii) do not exhibit increased extracellular concentration of adenosine *in vivo* (Fujita et al., 2014). We have not directly tested whether adenosine plays a role in the astrocytic modulation of synaptic GluN2B currents; however, using wild-type neurons and astrocytes, we have not observed a significant increase in the normalized surface expression of GluN2A and GluN2B subunits in mixed relative to neuronal cultures (Figures 8, 10). Our findings thus confirm that astrocytes regulate neuronal NMDAR channels, but suggest that the underlying mechanism does not involve changes in NMDAR subunit trafficking.

NMDAR currents are regulated by PKC in a complex manner; PKC-mediated NMDAR potentiation has been observed most often (Xiong et al., 1998; Lu et al., 1999; Zheng et al., 1999; Liao et al., 2001), but PKC-mediated suppression has also been reported (Markram and Segal, 1992). In the best studied form of PKC-mediated NMDAR current regulation, activation of G_q_ G protein-coupled (i.e., muscarinic M1 and glutamate mGluR5) receptors leads to activation of the phospholipase C/PKC/Src kinase signaling cascade and enhancement of NMDA-evoked whole-cell currents that is greater at the peak than at the plateau (Lu et al., 1999, 2000). The preferential enhancement of peak NMDAR currents was originally interpreted as PKC regulation of Ca^2+^-dependent inactivation (Lu et al., 1999), but is now understood as selective potentiation of GluN2A-containing NMDARs, which preferentially contribute to the peak NMDA-evoked currents (Yang et al., 2014). G_q_/PKC/Src-mediated regulation of GluN2A NMDARs is thus distinct from the astrocyte-induced potentiation of GluN2B NMDARs reported in the current study, although both events seem to be mediated by a change in the single channel kinetics rather than a change in the single channel conductance [(Lu et al., 1999) and Figure 8]. It is not entirely clear why the PKC-mediated modulation of GluN2B NMDARs has not been observed in the earlier studies; however, it is worth noting that the previous work was performed in the absence of glia, largely using heterologous expression systems or acutely dissociated neurons. It is possible that astrocyte-secreted factors responsible for GluN2B NMDAR regulation activate PKC through a non-canonical, G_q_ G protein-independent mechanism that is yet to be elucidated.

What is the molecular target of PKC phosphorylation? Although both GluN1 and GluN2 subunits contain multiple PKC phosphorylation consensus sites (Chen and Roche, 2007), mutation or deletion of these sites does not completely eliminate PKC-mediated NMDAR current enhancement, suggesting that PKC effects are at least partly indirect (i.e., mediated by phosphorylation of other neuronal proteins). The C terminus of the GluN2B subunit contains two adjacent Ser residues (Ser1303 and Ser1323) that can be phosphorylated by PKC (Chen and Roche, 2007); Ser1303 is also phosphorylated by calcium/calmodulin-dependent protein kinase II (CaMKII) (Omkumar et al., 1996). In *Xenopus* oocytes, mutations of Ser1303 and Ser1323 strongly attenuate, but do not completely block, the PKC modulation of recombinant GluN1/2B channels (Liao et al., 2001). We have not observed any difference in the phosphorylation status of Ser1303 between the two culture conditions either at baseline or following treatment with the PKC inhibitor GFX (Figure 10), indicating (i) that this residue is not involved in the astrocyte- and PKC-mediated modulation of synaptic GluN2B receptors and (ii) that in the intact neuronal milieu a kinase other than PKC (presumably CaMKII) has a dominant effect on Ser1303 phosphorylation. Additional work will be required to establish whether astrocyte-induced PKC activation leads to phosphorylation of a different residue within the GluN2B subunit or acts through an ancillary protein that is part of the NMDAR signaling complex (Figure 11). Notably, astrocytic regulation is restricted to synaptic GluN2B receptors (Figures 6, 7); given that GluN2B receptors can move between synaptic and extrasynaptic sites (Groc et al., 2006), it is not currently clear how this specificity is achieved. Most likely, kinases, phosphatases, and other signaling proteins that make up the supramolecular NMDA receptor complex differ between synaptic and extrasynaptic sites, and the differences in this subcellular environment play a role in the regulation of NMDAR channel activity.

What is the functional significance of synaptic GluN2B receptor regulation by astrocytes? At the early stages of postnatal development, there is a change in the neuronal NMDAR composition: GluN2B-containing NMDARs, which are the main NMDAR subtype expressed at birth, are partially replaced by GluN2A-containing NMDARs, which have a lower glutamate affinity and exhibit faster inactivation kinetics (Paoletti et al., 2013). This GluN2A-2B switch coincides with synaptic maturation; however, synapses with high GluN2A to GluN2B ratio require stronger stimulation to undergo long term potentiation (LTP; Paoletti et al., 2013). Thus, the GluN2A-2B switch is likely to have a negative effect on learning and memory. In agreement with this hypothesis, overexpression of the GluN2B subunit in the adult forebrain leads to more robust LTP and enhanced performance on various learning and memory tasks in both mice (Tang et al., 1999) and rats (Wang et al., 2009), even at an old age (Cao et al., 2007). Of greater physiological relevance, transgenic mice with high serum levels of the anti-aging factor klotho show an increase in the GluN2B portion of synaptic NMDAR currents, which is accompanied by enhanced LTP and better performance on tests of learning and memory; in humans, heterozygosity for a lifespan-extending variant of the *KLOTHO* gene is associated with increased klotho serum levels and better cognition (Dubal et al., 2014). While klotho appears to regulate the number of GluN2B receptors expressed at synapses (Dubal et al., 2014), a similar learning enhancement would be expected to result from an astrocyte-mediated increase in the activity of GluN2B NMDARs that are already present at postsynaptic sites. We therefore propose that astrocytes can dynamically regulate the synaptic GluN2A/2B current ratio and the threshold for LTP induction; identification of the astrocyte-secreted factor(s) that mediate(s) NMDAR regulation will enable the direct testing of this hypothesis.

Ca^2+^ influx through synaptic NMDARs leads to activation of FOXO, C/EBPβ, and AP-1 signaling pathways that together have a neuroprotective effect (Papadia et al., 2008). In contrast, activation of extrasynaptic NMDARs is generally neurotoxic (Hardingham et al., 2002). GluN2A- and GluN2B-containing NMDARs were originally hypothesized to preferentially signal to cell survival and cell death cascades, respectively (Liu et al., 2007); however, later studies have demonstrated that either receptor subtype can activate both neuroprotective and neurotoxic signaling pathways (von Engelhardt et al., 2007; Martel et al., 2009). More recently, it has been shown that the C terminus of GluN2B is more effective than the C terminus of GluN2A in promoting neuronal death; however, this effect was observed when both synaptic and extrasynaptic NMDARs were chronically activated (Martel et al., 2012). In contrast, trans-synaptic (physiologic) activation of GluN2B receptors was shown to be neuroprotective (Papadia et al., 2008; Martel et al., 2009). Interestingly, we have found that astrocyte-secreted factor(s) selectively increase the current flow through synaptic, but not extrasynaptic, GluN2B-containing NMDARs. In the setting of physiologic synaptic activity, the astrocyte-mediated increase in the synaptic GluN2B receptor activity would thus be expected to have a net neuroprotective effect.

In summary, we have shown that astrocyte-secreted soluble factor(s) can fine-tune the activity of synaptic GluN2B NMDAR channels. Through this presumably rapid and reversible PKC-mediated molecular mechanism, astrocytes can regulate synaptic AMPAR/NMDAR and GluN2A/2B ratios without altering the expression or trafficking of NMDAR channel subunits. Elucidating the details of the signal transduction pathway that mediates astrocytic regulation of synaptic GluN2B channels thus has a potential to foster the development of pharmacologic treatments that will enhance cognitive performance while strengthening neuroprotective signaling.

## Conflict of Interest Statement

The authors declare that the research was conducted in the absence of any commercial or financial relationships that could be construed as a potential conflict of interest.

## Footnotes

^1^http://rsbweb.nih.gov/ij/
